# Vascular Endothelial Growth Factor/Angiopoietin‐2 Bispecific Antibody‐Induced Vascular Normalization: From Basic Mechanisms to Therapeutic Applications in Breast Cancer

**DOI:** 10.1002/iid3.70408

**Published:** 2026-04-20

**Authors:** Meng Li, Fengjuan Han, Shuang Ling, Xiaoyue Su, Yuxin Zhao, Shaowa Lv, Xiwu Zhang

**Affiliations:** ^1^ Department of Traditional Chinese Medicine Heilongjiang University of Chinese Medicine Harbin City Heilongjiang Province China

**Keywords:** angiopoietin‐2, antiangiogenic therapy, bispecific antibody, breast cancer, tumor vasculature, vascular normalization, VEGF

## Abstract

Abnormal tumor vasculature is a hallmark of breast cancer, characterized by tortuous, hyperpermeable, and poorly perfused vessels that contribute to hypoxia, therapeutic resistance, and immune exclusion. Vascular endothelial growth factor (VEGF) and angiopoietin‐2 (ANG2) are central drivers of these vascular abnormalities, with VEGF promoting endothelial proliferation and leakiness, and ANG2 destabilizing vessel structure by antagonizing Tie2‐mediated pericyte coverage. Monotherapies targeting VEGF provide only transient vascular normalization, as compensatory ANG2 upregulation limits efficacy. Dual inhibition of VEGF and ANG2 via bispecific antibodies offers a mechanistically grounded strategy to achieve more durable vascular normalization. Preclinical studies demonstrate that VEGF/ANG2 bispecific antibodies enhance pericyte coverage, reduce leakage, improve perfusion, alleviate hypoxia, increase intratumoral drug delivery, and promote immune cell infiltration, thereby suppressing tumor growth and metastasis. Early clinical trials indicate acceptable safety profiles and biologic activity, with greatest therapeutic potential observed in combination with chemotherapy, immune checkpoint inhibitors, and radiotherapy. Ongoing translational studies are focused on optimizing dosing, identifying predictive biomarkers, and overcoming resistance mechanisms. This review summarizes the biology of VEGF and ANG2 signaling, mechanisms of vascular normalization, preclinical and clinical evidence of VEGF/ANG2 bispecific antibodies, and current and future therapeutic applications in breast cancer, highlighting their potential to enhance multimodal treatment efficacy and improve patient outcomes.

## Graphical abstract

VEGF/ANG2 bispecific antibody‐induced vascular normalization in breast cancer.

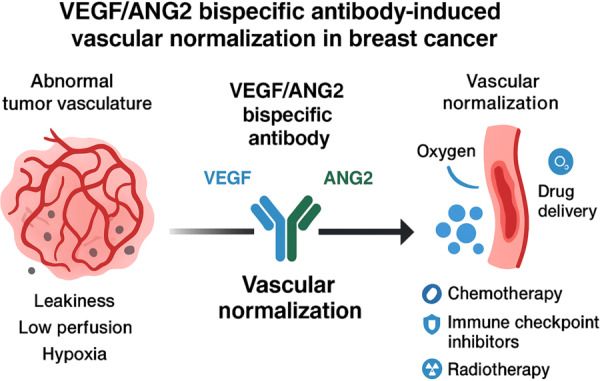

## 1 Introduction

Breast cancer remains the most prevalent malignancy among women worldwide and a leading cause of cancer‐related mortality [1, 2, 3]. Despite significant progress in early detection and multimodal therapy—including surgery, chemotherapy, radiotherapy, targeted therapy, and immunotherapy—therapeutic resistance and tumor recurrence continue to pose formidable challenges. Increasing evidence indicates that tumor vasculature is a central determinant of disease progression and treatment efficacy [4, 5]. Unlike normal vessels, breast cancer vasculature is highly aberrant, characterized by tortuous and dilated vessels, poor pericyte coverage, hyperpermeability, and heterogeneous perfusion. Direct experimental studies in breast cancer and solid tumor models have confirmed these features, including disrupted endothelial junctions, pericyte loss, and perfusion heterogeneity [6, 7]. These structural and functional abnormalities create a hostile tumor microenvironment (TME) characterized by hypoxia, elevated interstitial fluid pressure, metabolic reprogramming, and immune exclusion, which collectively compromise the delivery and effectiveness of systemic therapies [8, 9, 10, 11].

VEGF and ANG2 are key drivers of these vascular abnormalities, acting through complementary mechanisms. VEGF promotes endothelial proliferation, sprouting angiogenesis, and vascular leakiness, whereas ANG2 antagonizes Tie2‐mediated pericyte stabilization, thereby preventing vessel maturation [12, 13, 14, 15, 16]. The interplay of these pathways is particularly relevant in aggressive breast cancer subtypes such as triple‐negative and HER2‐positive tumors, which exhibit high VEGF/ANG2 expression and correlate with hypoxia, metastasis, and poor prognosis [17, 18, 19]. Anti‐VEGF monotherapy has been shown to transiently normalize tumor vessels; however, compensatory upregulation of ANG2 frequently limits the durability of this effect, highlighting the need for dual‐targeted strategies [15, 20, 21, 22].

VEGF/ANG2 bispecific antibodies have emerged as a promising approach to achieve durable vascular normalization. Preclinical studies demonstrate that simultaneous blockade of both pathways enhances pericyte coverage, reduces vascular leakiness, improves perfusion, alleviates hypoxia, and facilitates infiltration of cytotoxic T cells in advance solid tumors [23, 24, 25, 26]. These vascular improvements potentiate chemotherapeutic drug delivery, increase radiosensitivity, and enhance response to immune checkpoint inhibitors, effectively overcoming barriers imposed by the abnormal TME. Early‐phase clinical trials, including studies with vanucizumab, indicate acceptable safety profiles and biologic activity, particularly when combined with chemotherapy or immunotherapy [27, 28, 29, 30, 31].

Despite these advances, several critical gaps remain. First, the optimal integration of VEGF/ANG2 bispecific antibodies into multimodal treatment regimens remains undefined, including timing, dosing, and combination strategies with chemotherapy, immunotherapy, radiotherapy, or molecularly targeted agents. Second, predictive biomarkers for patient selection, such as circulating ANG2 levels, tumor perfusion imaging, and gene expression signatures, are still under investigation and require validation in larger cohorts. Third, adaptive resistance mechanisms, including activation of alternative angiogenic pathways (e.g., FGF, PDGF), may limit long‐term efficacy. Addressing these gaps is essential for translating vascular normalization into durable clinical benefit.

In this review, we synthesize current understanding of breast cancer vasculature, emphasizing the pathological features and clinical implications of VEGF‐ and ANG2‐driven vascular abnormalities. We provide an in‐depth overview of preclinical and early clinical evidence supporting VEGF/ANG2 bispecific antibodies and discuss their potential to enhance multimodal therapy efficacy. By highlighting unresolved questions and emerging translational strategies, this review aims to provide a comprehensive framework for optimizing vascular‐targeted therapy and guiding future research in breast cancer.

## 2 Breast Cancer Vasculature: Pathological Features and Clinical Implications

Breast cancer vasculature exhibits profound structural and functional abnormalities compared with normal tissue vessels, forming the pathological foundation for tumor progression, metastatic dissemination, and therapeutic resistance. According to Jain's vascular normalization hypothesis, the tumor endothelium is characterized by chaotic organization and deregulated angiogenic signaling, which together create a hostile TME [32, 33, 34, 35]. Antiangiogenic therapy can transiently remodel these abnormal vessels toward a more organized and functional state, a process termed vascular normalization. During this normalization window, pericyte coverage increases, endothelial junctions tighten, vessel leakiness decreases, and perfusion and oxygen delivery are improved. This temporary restoration of vascular structure enhances the delivery of chemotherapeutic drugs and facilitates immune cell infiltration. The duration and extent of the normalization window are dose‐ and context‐dependent, highlighting the importance of optimizing antiangiogenic therapy schedules to maximize therapeutic benefit. Breast cancer subtypes exhibit distinct vascular phenotypes and angiogenic profiles. ER‐positive tumors tend to show relatively organized vasculature with moderate VEGF expression, whereas HER2‐positive tumors display higher microvessel density and increased VEGF and ANG2 expression, contributing to more chaotic vascular networks [36, 37]. Triple‐negative breast cancer (TNBC) is characterized by highly aberrant vasculature, including pericyte deficiency, increased vessel permeability, and elevated pro‐angiogenic signaling, which collectively hinder immune cell infiltration and promote hypoxia‐driven progression [38, 39]. These primary studies demonstrate how molecular subtype dictates tumor vascular architecture and angiogenic activity, providing mechanistic insights relevant for targeted anti‐angiogenic therapies.

### 2.1 Structural Abnormalities

Tumor blood vessels in breast cancer are highly tortuous, dilated, and irregular in diameter, often lacking the hierarchical organization of arterioles, capillaries, and venules. Pericyte coverage is sparse or loosely attached, resulting in fragile vessels prone to leakage [7, 40, 41]. Endothelial cells (ECs) are disorganized and exhibit widened intercellular junctions due to aberrant expression of tight‐junction proteins such as VE‐cadherin and claudins [42, 43, 44]. This structural immaturity leads to impaired vessel integrity and unbalanced perfusion, with areas of hyperperfusion juxtaposed with hypoxic regions. Imaging studies have revealed that breast tumors often display heterogeneous perfusion patterns on dynamic contrast‐enhanced MRI, reflecting these underlying microvascular defects [45, 46].

### 2.2 Functional Abnormalities

**Figure 1:**
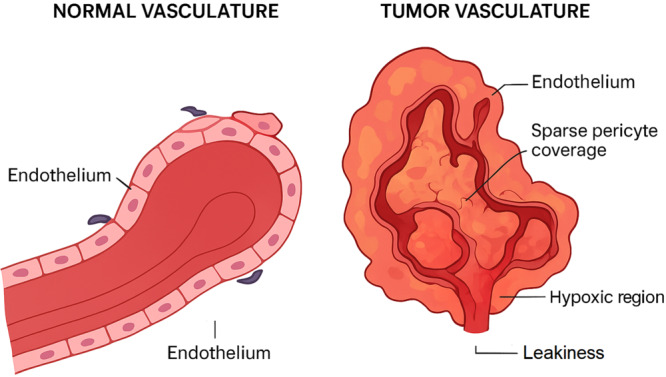
Comparison of normal vasculature with tumor vasculature.

Functionally, these structural abnormalities translate into hyperpermeable and inefficient vasculature. VEGF‐driven fenestrations combined with poor pericyte support cause excessive plasma protein leakage and elevated interstitial fluid pressure (IFP), which impairs drug delivery. Hypoxia arising from inefficient oxygen transport activates hypoxia‐inducible factors (HIF‐1α and HIF‐2α), which in turn upregulate VEGF, ANG2, and CXCL12, reinforcing the angiogenic switch in a feed‐forward loop. Hypoxia also shifts cancer cell metabolism toward glycolysis (the “Warburg effect”) and drives epithelial–mesenchymal transition (EMT), enhancing invasiveness [47, 48, 49, 50]. Collectively, these functional defects foster therapeutic resistance and immune evasion, highlighting the need for vascular normalization strategies to restore perfusion, reduce hypoxia, and improve drug and immune cell delivery [51] (Figure 1).

### 2.3 Immune Implications of Abnormal Vasculature

CD31 expressed on endothelial cells facilitates CD8 + T cell rolling and migration into the tumor parenchyma via the rolling circle model [52]. Aberrant tumor vasculature, characterized by disorganized endothelial cells and disrupted junctions, impairs this process, limiting T cell infiltration into the tumor core. Furthermore, hypoxia and VEGF‐driven signaling upregulate chemokines such as CXCL12, which recruit immunosuppressive cells including regulatory T cells and myeloid‐derived suppressor cells (MDSCs) [11], while concurrently reducing the activity of effector CD8+ T cells. Collectively, these vascular and chemokine‐mediated mechanisms contribute to immune evasion and therapeutic resistance within the tumor microenvironment.

### 2.4 Clinical Implications

The clinical manifestations of vascular abnormalities in breast cancer are multifaceted [53]. First, impaired perfusion and hypoxia are strongly correlated with poor prognosis and resistance to radiotherapy and chemotherapy. Second, microvessel density (MVD), particularly when driven by VEGF/ANG2 signaling [20], has been associated with higher histological grade, increased risk of recurrence, and reduced overall survival in breast cancer patients. Third, imaging biomarkers such as dynamic contrast‐enhanced MRI (DCE‐MRI) and contrast‐enhanced ultrasound have been utilized to non‐invasively evaluate tumor vascular heterogeneity, serving as predictive indicators of therapeutic response [31]. Importantly, the recognition of vasculature‐driven therapeutic resistance has motivated the integration of vascular‐targeting strategies with chemotherapy, radiotherapy, endocrine therapy, and immunotherapy in breast cancer management.

In summary, the abnormal vasculature in breast cancer not only underpins tumor growth and metastatic spread but also establishes a major barrier to treatment efficacy. This dual role—facilitating disease progression while undermining therapy—provides a compelling rationale for vascular normalization strategies, particularly those targeting VEGF and ANG2 pathways, which will be discussed in subsequent sections.

## 3 Mechanisms and Preclinical Evidence

The therapeutic rationale for dual blockade of VEGF and ANG2 lies in their synergistic regulation of tumor angiogenesis and vascular remodeling. Preclinical studies have demonstrated that inhibiting VEGF alone induces transient vascular normalization, but this effect is often short‐lived due to compensatory upregulation of ANG2. In mouse models of breast cancer, combined inhibition of VEGF and ANG2 has been shown to significantly reduce vessel density, normalize vessel structure, and enhance perfusion, thereby restoring oxygen delivery and reducing hypoxia‐inducible signaling antibodies aim to achieve more durable normalization, enhanced perfusion, and improved drug delivery in breast cancer.

### 3.1 VEGF Signaling in Breast Cancer Angiogenesis

VEGF is a master regulator of tumor angiogenesis and is highly expressed in breast cancer, particularly in triple‐negative and HER2‐positive subtypes. Binding of VEGF‐A to VEGFR2 on ECs promotes proliferation, migration, and sprouting angiogenesis [12, 15]. VEGF also induces fenestrations and reduces tight‐junction protein expression, driving vascular permeability. Experimental overexpression of VEGF in breast cancer cell lines leads to hypervascular and hypoxic tumors, while VEGF inhibition via bevacizumab or genetic silencing decreases microvessel density (MVD) but frequently triggers adaptive resistance through ANG2 upregulation [20].

### 3.2 ANG2 Signaling and Vascular Destabilization

**Figure 2:**
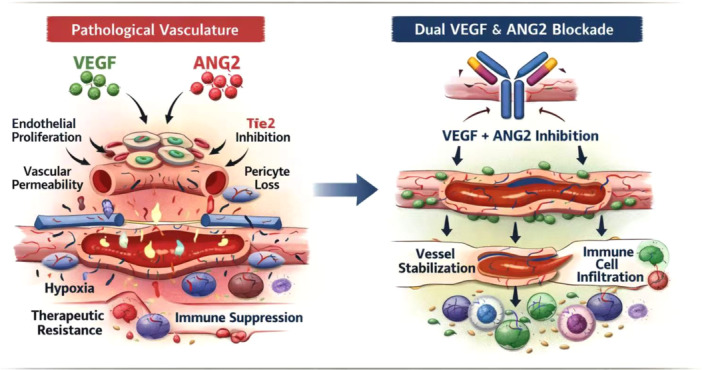
Mechanistic interplay of VEGF and ANG2 signaling in abnormal vasculature. Illustration of VEGF‐driven endothelial proliferation and permeability, together with ANG2‐mediated antagonism of Tie2 signaling and pericyte detachment. The combined effect generates immature, unstable vessels with enhanced leakiness and hypoxia, contributing to therapeutic resistance and immune suppression. Dual blockade of VEGF and ANG2 via bispecific antibodies restores vascular stability, improves perfusion, and promotes immune cell infiltration.

ANG2 acts primarily through the Tie2 receptor on ECs and pericytes. Unlike ANG1, which stabilizes vessels, ANG2 is context‐dependent: in the presence of VEGF, it promotes angiogenesis and vascular sprouting; in its absence, it induces vessel regression. In breast cancer, ANG2 expression is upregulated by hypoxia and inflammatory cytokines such as TNF‐α. Elevated ANG2 levels correlate with high‐grade tumors, nodal metastasis, and poor overall survival [54]. Mechanistically, ANG2 disrupts endothelial–pericyte interactions, destabilizing the vasculature and creating permissive niches for tumor cell intravasation and metastasis. The interplay between VEGF and ANG2 signaling is central to the maintenance of vascular abnormalities in breast cancer. VEGF‐A binding to VEGFR2 activates downstream pathways such as PI3K/AKT and MAPK/ERK, leading to endothelial proliferation, migration, and vascular leakiness, while stabilizing hypoxia‐inducible factor‐1α (HIF‐1α) signaling. In parallel, ANG1 normally binds to Tie2 to promote vessel stability through pericyte recruitment. However, ANG2 functions as a context‐dependent antagonist of Tie2: in the presence of VEGF, it accelerates angiogenesis, whereas in its absence it induces vascular regression [55]. Together, VEGF and ANG2 synergize to generate immature, leaky vasculature and foster an immunosuppressive milieu characterized by reduced CD8⁺ T‐cell infiltration and increased regulatory T cells and myeloid‐derived suppressor cells (MDSCs). As depicted in Figure 2, this dual signaling convergence explains the rationale for bispecific antibodies that simultaneously block VEGF and ANG2 to restore vascular stability and enhance therapeutic response.

As depicted in Figure 2, this dual signaling convergence explains the rationale for bispecific antibodies that simultaneously block VEGF and ANG2 to restore vascular stability and enhance therapeutic response.

### 3.3 Synergistic Role of VEGF and ANG2 in Abnormal Vasculature

The VEGF and ANG2 pathways converge to exacerbate vascular abnormalities. VEGF‐driven angiogenesis generates immature, leaky vessels, while ANG2 destabilizes endothelial‐pericyte junctions, preventing vessel maturation. This synergistic action results in chaotic vascular networks characterized by poor perfusion and hypoxia [56, 57, 58]. In mouse models of breast cancer, combined inhibition of VEGF and ANG2 has been shown to significantly reduce vessel density, normalize vessel structure, and enhance perfusion, thereby restoring oxygen delivery and reducing hypoxia‐inducible signaling [59, 60, 61]. Importantly, this normalization window allows improved efficacy of concomitant therapies such as chemotherapy, radiotherapy, and immune checkpoint inhibitors.

### 3.4 Preclinical Evidence of VEGF/ANG2 Bispecific Antibodies

Several bispecific antibody constructs have been developed to simultaneously inhibit VEGF and ANG2, including A2V and vanucizumab. In xenograft and orthotopic breast cancer models, these bispecific antibodies demonstrated superior antitumor activity [59, 62, 63]. Preclinical data consistently showed that treatment with dual‐targeting antibodies led to enhanced vascular normalization, manifested by improved pericyte coverage, reduced vessel tortuosity, and decreased leakage, thereby generating more stable and functional vasculature. At the same time, perfusion and oxygenation were significantly improved, alleviating tumor hypoxia and suppressing hypoxia‐inducible signaling [64, 65, 66]. These vascular improvements also translated into enhanced delivery and therapeutic efficacy of chemotherapeutic agents, including doxorubicin and paclitaxel, as well as nanoparticle‐based agents, in preclinical models of breast and other solid tumors [67, 68, 69]. Furthermore, dual VEGF/ANG2 inhibition reprogrammed the tumor microenvironment, improving immune cell infiltration and limiting the recruitment of immunosuppressive myeloid‐derived suppressor cells (MDSCs) in preclinical tumor models [70]. Importantly, metastasis studies in mouse models indicated that VEGF/ANG2 blockade decreased metastatic spread to distant organs, suggesting that dual inhibition provides benefits beyond local tumor control in various solid tumors [71, 72, 73].

### 3.5 Insights From Translational Research

Although most evidence is derived from animal models, translational studies using patient‐derived xenografts (PDX) and organotypic cultures of breast cancer tissue provide further support. Analysis of circulating ANG2 and VEGF levels in breast cancer patients revealed dynamic changes during anti‐VEGF therapy, with compensatory elevation of ANG2 contributing to resistance [54]. These findings underscore the rationale for dual‐targeting approaches in clinical settings.

In summary, preclinical studies strongly support VEGF/ANG2 bispecific antibodies as a means to achieve durable vascular normalization in breast cancer. By simultaneously suppressing two central angiogenic drivers, these agents can overcome the limitations of monotherapy and establish a microenvironment more conducive to effective systemic therapies.

## 4 Therapeutic Applications in Breast Cancer

**Figure 3:**
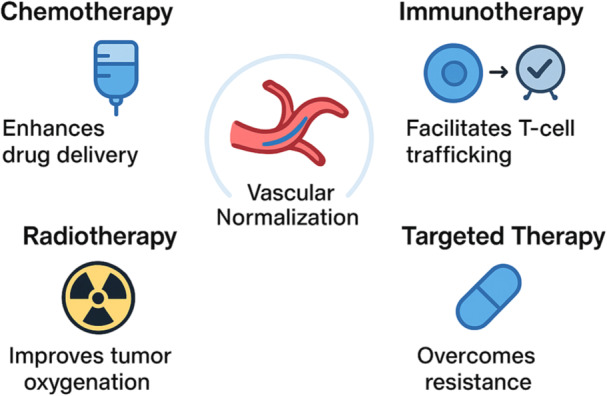
Therapeutic strategies of VEGF/ANG2 bispecific antibodies in breast cancer. Overview of potential applications of VEGF/ANG2 bispecific antibodies in multimodal therapy. Vascular normalization enhances chemotherapy drug delivery, improves tumor oxygenation for radiotherapy, and facilitates cytotoxic T‐cell trafficking to potentiate immune checkpoint inhibitors. Integration with targeted therapies (e.g., HER2 or PI3K/AKT inhibitors) may further overcome angiogenesis‐driven resistance, highlighting the versatility of bispecific antibodies in breast cancer treatment.

The preclinical evidence supporting VEGF/ANG2 bispecific antibodies has catalyzed the exploration of their clinical applications in breast cancer. Given the complexity of tumor angiogenesis and the multifaceted role of abnormal vasculature in therapeutic resistance, dual blockade strategies are being investigated across several treatment contexts, including as monotherapy, in combination with cytotoxic chemotherapy, and in integration with immunotherapy [59, 67, 68]. These vascular improvements potentiate chemotherapeutic drug delivery, increase radiosensitivity, and enhance response to immune checkpoint inhibitors, effectively overcoming barriers imposed by the abnormal TME (Figure 3).

### 4.1 Monotherapy and Early Clinical Experience

VEGF/ANG2 bispecific antibodies, such as vanucizumab, have undergone phase I and II clinical evaluation across solid tumors, including subsets of advanced breast cancer. Initial results demonstrated acceptable safety profiles, with manageable adverse events primarily consisting of hypertension, proteinuria, and gastrointestinal toxicities similar to VEGF‐targeted therapies [27]. Importantly, pharmacodynamic analyses indicated effective suppression of circulating VEGF and ANG2 levels, along with imaging evidence of vascular remodeling. However, clinical efficacy as monotherapy was modest, with partial responses and disease stabilization reported in a fraction of patients. These findings reinforce the notion that bispecific antibodies are most effective when combined with complementary therapeutic modalities.

### 4.2 Combination With Chemotherapy

Cytotoxic chemotherapy remains a cornerstone of breast cancer management, particularly in triple‐negative breast cancer (TNBC). Yet, its efficacy is limited by poor intratumoral drug delivery due to high interstitial fluid pressure and heterogeneous perfusion. By promoting vascular normalization, VEGF/ANG2 blockade enhances drug penetration into tumor tissues. Preclinical breast cancer models demonstrated significantly improved efficacy of doxorubicin and paclitaxel when administered in combination with VEGF/ANG2 bispecific antibodies compared with chemotherapy alone [67, 68]. Early‐phase clinical trials have echoed these findings, with reports of increased objective response rates and progression‐free survival in patients treated with vanucizumab plus paclitaxel. Importantly, these benefits were most pronounced in patients with high baseline levels of circulating ANG2, suggesting the potential for biomarker‐driven patient stratification.

### 4.3 Integration With Immunotherapy

Immune checkpoint inhibitors (ICIs) have emerged as promising therapies for TNBC, yet their clinical benefit is limited by immune exclusion and immunosuppressive TMEs. VEGF and ANG2 not only drive angiogenesis but also contribute to immune suppression by reducing T‐cell infiltration and enhancing recruitment of regulatory T cells and MDSCs. Dual blockade has been shown in preclinical breast cancer models to enhance the efficacy of anti–PD‐1/PD‐L1 therapy by reprogramming the vasculature into a permissive interface for T‐cell trafficking. Early clinical observations indicate that VEGF/ANG2 inhibition can enhance the efficacy of immune checkpoint inhibitors in cancer patients by normalizing tumor vasculature, improving T cell infiltration, and reducing immunosuppressive myeloid cell populations [59, 74]. Several ongoing clinical trials are currently evaluating VEGF/ANG2 bispecific antibodies in combination with checkpoint blockade, which may expand immunotherapy applicability in breast cancer beyond current responders.

### 4.4 Potential Synergy With Radiotherapy and Targeted Therapies

Radiotherapy efficacy depends heavily on adequate tumor oxygenation, which is often compromised by disorganized vasculature. Preclinical evidence suggests that VEGF/ANG2 dual inhibition can alleviate hypoxia, thereby sensitizing glioma to radiation‐induced DNA damage [75]. Moreover, the intersection between angiogenic signaling and growth factor pathways such as HER2 and PI3K/AKT raises the possibility of combining VEGF/ANG2 bispecific antibodies with molecularly targeted agents. This combinatorial approach could be particularly relevant in HER2‐positive and PIK3CA‐mutated breast cancers, where angiogenesis contributes to therapeutic resistance.

### 4.5 Clinical Challenges and Translational Considerations

Despite promising preclinical and early clinical findings, several challenges remain. First, the identification of predictive biomarkers is essential to guide patient selection, as not all breast cancer subtypes or patients derive equal benefit. Circulating ANG2 levels, tumor perfusion imaging, and gene expression signatures are under investigation as potential biomarkers [54, 76]. Second, the risk of cumulative toxicity when combining bispecific antibodies with chemotherapy or immunotherapy requires careful dose optimization and patient monitoring. Third, resistance mechanisms, including upregulation of alternative angiogenic factors such as FGF and PDGF, may limit long‐term efficacy, underscoring the need for rational combination regimens.

In summary, VEGF/ANG2 bispecific antibodies represent a versatile therapeutic platform with broad applications in breast cancer. Their greatest promise lies in combination strategies, particularly with chemotherapy and immunotherapy, where they have the potential to overcome vascular and immune barriers to treatment. Ongoing clinical trials will be critical in defining their role within standard‐of‐care regimens and determining whether they can deliver durable clinical benefit across diverse breast cancer subtypes.

### 4.6 Clinical Translation and Therapeutic Applications

The transition of VEGF/ANG2 bispecific antibodies from preclinical discovery to clinical evaluation has been spearheaded by molecules such as vanucizumab (a CrossMab format targeting VEGF‐A and ANG2) and BI 836880 (a Nanobody® construct). In a first‐in‐human trial (NCT01688206), vanucizumab demonstrated a tolerable safety profile, with class‐related toxicities similar to VEGF inhibitors, including hypertension and proteinuria. Pharmacodynamic studies confirmed rapid depletion of free VEGF‐A and ANG2, although clinical efficacy as a monotherapy remained modest [27]. The phase II McCAVE study in metastatic colorectal cancer (NCT02141295) compared vanucizumab plus FOLFOX to bevacizumab plus FOLFOX. While safety was manageable, no significant progression‐free survival (PFS) benefit was observed, highlighting the challenge of surpassing established VEGF inhibitors in large‐scale trials [77].

Parallel efforts by Boehringer Ingelheim evaluated BI 836880 in advanced solid tumors (NCT02674152), where dose‐escalation studies established a manageable safety profile and pharmacodynamic biomarker modulation, including sustained decreases in circulating VEGF and ANG2. Building on these findings, a combination study of BI 836880 with the anti–PD‐1 antibody ezabenlimab (NCT03468426) enrolled 133 patients with advanced solid tumors. The regimen was generally tolerable, and early evidence of immune reprogramming and clinical activity was reported, supporting the hypothesis that vascular normalization enhances immunotherapy responses [29].

In addition to these clinical programs, preclinical constructs such as the A2V CrossMAb have provided strong mechanistic evidence of dual blockade efficacy. In breast cancer and glioblastoma xenografts, A2V promoted durable vascular normalization, reduced hypoxia, enhanced chemotherapy penetration, and facilitated CD8+ T‐cell infiltration while suppressing immunosuppressive myeloid populations [73]. Similarly, ANG2‐blocking antibodies such as LC06 reduced metastatic spread by stabilizing endothelial barriers in lung and breast cancer models [62]. These data reinforce the notion that VEGF/ANG2 dual blockade achieves biological effects unattainable by monotherapies.

**Figure 4:**
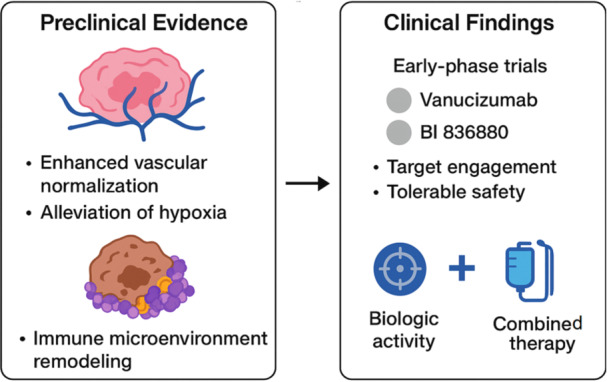
Clinical translation of VEGF/ANG2 bispecific antibodies. Summary of representative preclinical and clinical findings. Preclinical models demonstrate enhanced vascular normalization, alleviation of hypoxia, and immune microenvironment remodeling. Early‐phase trials (e.g., vanucizumab and BI 836880) confirm target engagement, tolerable safety, and biologic activity, with the greatest promise in rational combinations with chemotherapy, radiotherapy, and immunotherapy.

A summary of representative clinical and translational studies is provided in Table 1, highlighting key trial identifiers, endpoints, and sample sizes. Together with the schematic in Figure 4, these findings illustrate the therapeutic rationale for VEGF/ANG2 bispecific antibodies.

**Table 1:** Clinical and translational studies of VEGF/ANG2 bispecific antibodies in solid tumors.

Antibody (construct)	Developer	Trial ID (NCT)	Phase/Sample size	Indication	Primary endpoints	Key findings	References
Vanucizumab (CrossMab anti‐VEGF‐A/anti‐Ang‐2)	Roche/Genentech	NCT01688206	Phase I, *n* = 56	Advanced solid tumors	Safety, PK/PD	Manageable safety; class‐related VEGFi toxicities. PD: ↓free VEGF‐A and Ang‐2. Limited monotherapy efficacy.	[27]
Vanucizumab ± FOLFOX (McCAVE study)	Roche	NCT02141295	Phase II, *n* ≈ 200	Metastatic colorectal cancer	PFS	No PFS benefit vs. bevacizumab; acceptable safety.	[77]
BI 836880 (VEGF/Ang‐2 Nanobody®)	Boehringer Ingelheim	NCT02674152	Phase I dose‐escalation, *n* = 54	Advanced solid tumors	DLT, MTD, PK	Tolerable safety; biomarker‐confirmed target engagement.	[29]
BI 836880 + anti–PD‐1 (ezabenlimab)	Boehringer Ingelheim	NCT03468426	Phase I, *n* = 133	Advanced solid tumors	Safety, RP2D	Combination tolerable; preliminary efficacy; immune activation observed.	[78]
A2V CrossMAb (research construct)	Academic	Preclinical	Mouse/PDX models	Glioblastoma, breast cancer	Vascular normalization, survival	Improved pericyte coverage, ↓hypoxia, ↑immune infiltration, prolonged survival.	[73]
Ang‐2 blockade antibodies (e.g., LC06)	Academic/biotech	Preclinical	Mouse lung metastasis models	Breast/lung cancer	Vascular stability, metastasis	ANG2 inhibition ↓endothelial leakiness, ↓metastatic burden.	[62]

### 4.7 Summary of Clinical Translation

In summary, clinical experience with VEGF/ANG2 bispecific antibodies has thus far demonstrated biological activity and tolerability, but limited efficacy as monotherapies. Large‐scale studies such as the McCAVE trial confirmed manageable safety but failed to show superiority over standard VEGF inhibitors, underscoring the challenges of displacing entrenched anti‐VEGF therapies. However, pharmacodynamic and immunologic findings consistently support the notion that dual blockade achieves more durable vascular normalization and immune reprogramming compared with VEGF inhibition alone.

The growing body of evidence suggests that the true therapeutic potential of VEGF/ANG2 bispecific antibodies lies in combination strategies—particularly with chemotherapy, radiotherapy, and immune checkpoint inhibitors—where vascular normalization can synergize with cytotoxic or immune‐mediated mechanisms. Ongoing and future trials will be critical to define optimal combinations, biomarkers of response, and clinical settings where these agents can provide meaningful benefit, including in breast cancer. This translational trajectory sets the stage for a new generation of microenvironment‐targeted therapies, bridging angiogenesis inhibition with immuno‐oncology.

## 5 Conclusions and Future Perspectives

VEGF/ANG2 bispecific antibodies represent a promising advancement in the field of antiangiogenic therapy for breast cancer, offering a mechanistically grounded approach to address the limitations of VEGF monotherapy. Preclinical studies have convincingly demonstrated that dual blockade not only normalizes aberrant tumor vasculature but also improves perfusion, alleviates hypoxia, enhances drug delivery, and reprograms the immune microenvironment. These findings provide a strong rationale for integrating VEGF/ANG2 bispecific antibodies into multimodal therapeutic regimens.

Clinically, early‐phase trials indicate that VEGF/ANG2 bispecific antibodies are well‐tolerated and biologically active, although monotherapy responses are modest. The most compelling potential lies in combination strategies, particularly with cytotoxic chemotherapy and immune checkpoint inhibitors, where vascular normalization can overcome barriers to drug delivery and immune cell infiltration. Radiotherapy and targeted therapy combinations also hold promise, especially in subtypes characterized by angiogenesis‐driven therapeutic resistance. Importantly, the integration of predictive biomarkers—including circulating ANG2 levels, imaging‐based perfusion metrics, and molecular signatures—may enable personalized selection of patients most likely to benefit from dual blockade.

Despite these advances, several challenges remain. Resistance through alternative angiogenic pathways, optimal timing and dosing of combination therapies, and the management of cumulative toxicity are critical areas requiring further investigation. Additionally, longitudinal assessment of vascular and immune remodeling in patients will be essential to optimize treatment schedules and to identify robust biomarkers of response.

Looking forward, VEGF/ANG2 bispecific antibodies are likely to play a central role in next‐generation precision oncology strategies for breast cancer. Their ability to simultaneously modulate angiogenesis and the TME positions them as versatile agents capable of enhancing the efficacy of multiple treatment modalities. Continued translational research and well‐designed clinical trials are essential to define their optimal clinical use, overcome potential resistance mechanisms, and ultimately improve patient outcomes across diverse breast cancer subtypes.

## Author Contributions

**Meng Li:** writing – review and editing, writing – original draft, conceptualization, funding acquisition, investigation, methodology, validation, formal analysis, project administration, data curation, supervision, resources, software. **Fengjuan Han:** writing – review and editing, writing – original draft, conceptualization, investigation, methodology, validation, formal analysis, software. **Shuang Ling:** writing – review and editing, writing – original draft, conceptualization, investigation, methodology. **Xiaoyue Su:** writing – review and editing, writing – original draft, conceptualization, investigation, methodology. **Yuxin Zhao:** writing – review and editing, writing – original draft, conceptualization, investigation, methodology. **Shaowa Lv:** writing – review and editing, writing – original draft, investigation, methodology. **Xiwu Zhang:** writing – review and editing, writing – original draft, conceptualization, funding acquisition, investigation, methodology, validation, formal analysis, project administration, data curation, supervision, resources, software.

## Conflicts of Interest

The authors declare no conflicts of interest.

## Data Availability

The data that support the findings of this study are available from the corresponding author upon reasonable request.
